# Frontline evaluation: Atezolizumab–bevacizumab versus lenvatinib for BCLC stage B hepatocellular carcinoma exceeding the up‐to‐seven criteria

**DOI:** 10.1002/cam4.70217

**Published:** 2024-09-20

**Authors:** Masamichi Kimura, Koji Nishikawa, Jun Imamura, Kiminori Kimura

**Affiliations:** ^1^ Department of Hepatology, Tokyo Metropolitan Cancer and Infectious Diseases Center Komagome Hospital Bunkyo‐ku Tokyo Japan

**Keywords:** atezolizumab, Barcelona clinic liver cancer stage B, bevacizumab, hepatocellular carcinoma, lenvatinib

## Abstract

**Introduction:**

This study aimed to evaluate the efficacy and safety of atezolizumab combined with bevacizumab (Atez/Bev) compared to lenvatinib (LEN) as first‐line systemic therapy for patients with Barcelona Clinic Liver Cancer (BCLC) stage B hepatocellular carcinoma (HCC) exceeding the up‐to‐seven criteria threshold, who are typically unsuitable for transarterial chemoembolization (TACE).

**Methods:**

A retrospective analysis was conducted on 49 consecutive patients with HCC treated at Tokyo Metropolitan Komagome Hospital between May 2018 and October 2023. The patients were divided into two groups: the Atez/Bev group (21 patients) and the LEN group (28 patients). Eligibility criteria included Child‐Pugh A classification, no prior systemic therapy, and ineligibility for resection, ablation, or transplantation. Treatment outcomes were assessed through periodic imaging and laboratory tests, evaluating OS, PFS, ORR, and disease control rate (DCR).

**Results:**

Both groups demonstrated comparable baseline characteristics, with a median follow‐up of 15.4 months. No significant difference was observed in OS between the Atez/Bev and LEN groups (median OS: 19.80 vs. 22.20 months, *p* = 0.763). The median PFS was 10.23 months for Atez/Bev and 7.20 months for LEN (*p* = 0.343). There were no statistically significant differences in ORR or DCR between the two groups. Common adverse events included elevated AST and ALT levels, with no significant difference in the overall rate of adverse events between the groups.

**Conclusions:**

Atez/Bev and LEN demonstrated comparable efficacy and safety as first‐line systemic treatments for patients with BCLC stage B HCC exceeding the up‐to‐seven criteria. Both therapeutic options are viable for this population, though further large‐scale prospective studies are required to confirm these findings.

## 1 INTRODUCTION

Hepatocellular carcinoma (HCC) is a common malignant neoplasm.^1^ The vast majority (over 80%) of individuals with HCC are diagnosed when the disease has reached either an intermediate or advanced stage,^2^, ^3^ significantly affecting patient outcomes.^4^, ^5^, ^6^

According to Barcelona Clinic Liver Cancer (BCLC) standards, transarterial chemoembolization (TACE) is the main conventional approach for treating intermediate‐stage HCC.^7^ Nonetheless, the efficacy of TACE is compromised or deemed inappropriate for patients presenting with extensive and multifocal tumors, particularly those surpassing the up‐to‐seven threshold, thus necessitating multiple TACE interventions.^8^, ^9^ The up‐to‐seven benchmark refers to the sum of the number of tumors and maximum tumor diameter (cm) exceeding seven. In principle, TACE exacerbates tumor hypoxia and triggers a corresponding cellular response, potentially leading to the upregulation of both vascular endothelial growth factor (VEGF) and fibroblast growth factor (FGF), hence fostering tumor revascularization and advancement.^10^, ^11^ Furthermore, certain HCC cases may fail to absorb lipiodol, thereby diminishing the effectiveness of treatment.^12^ Hence, attaining a complete response (CR) to TACE, particularly when multiple sessions are indicated, along with desirable survival metrics, poses a substantial challenge for patients with advanced substages of intermediate HCC.^13^ The median survival of patients with different substages of intermediate HCC, namely substage 1 (B1), substage 2 (B2), and substage 3 (B3), has been reported to notably decline (44.8, 21.5, and 11.3 months, respectively, *p* < 0.001).^14^ In addition, a subset of patients may experience deterioration of their hepatic function following successive TACE treatments.^15^

Consequently, various systemic therapies have been investigated as primary treatments for intermediate‐stage HCC, particularly targeting larger (≥5 cm) or numerous tumors, especially those surpassing the up‐to‐seven threshold.^16^, ^17^ Atezolizumab combined with bevacizumab (Atez/Bev) and lenvatinib (LEN) have been endorsed as initial treatments for advanced HCC.^18^, ^19^ Notably, the synergy of atezolizumab, a programmed death‐ligand 1 (PD‐L1) blocker, with bevacizumab, an antagonist of VEGF, has been validated for its superior efficacy and safety over sorafenib monotherapy in the IMbrave150 trial.^20^ Lenvatinib, an emerging oral agent that targets multiple tyrosine kinases and disrupts both VEGF and FGF signaling pathways,^21^ has gained broad authorization as a primary treatment modality for inoperable HCC, as supported by the findings of the REFLECT study.^19^

In our study, we aimed to assess the efficacy and safety profiles of Atez/Bev and LEN as systemic treatment options for patients with intermediate‐stage HCC for whom TACE is deemed inappropriate based on the up‐to‐seven criteria.

## 2 MATERIALS AND METHODS

### 2.1 Study protocol

This study was approved by the ethics committee of the Tokyo Metropolitan Komagome Hospital (approval no. 2516) and adhered to the principles of the Declaration of Helsinki. The requirement for informed consent was waived owing to the retrospective nature of the study, which ensures no compromise to the welfare or rights of the patients. All data were anonymized to safeguard patient privacy. We conducted a retrospective review of the medical records of 178 sequential patients with HCC who underwent treatment at our institution. Specifically, patients treated with LEN were recruited between May 2018 and October 2023, whereas those receiving Atez/Bev were recruited from October 2020 to October 2023. This distinction is made to reflect the differing availability periods of the two treatment options, with Atez/Bev being introduced for HCC treatment at a later date than that of LEN.

### 2.2 Inclusion and exclusion criteria

Eligible patients met the following criteria: (a) HCC diagnosis confirmed by the European Association for the Study of the Liver (EASL) standards, without previous systemic therapy; (b) Child‐Pugh class A liver function, as required by the Japanese healthcare insurance system for the administration of Atez/Bev and LEN, ensuring patients have the necessary liver function for safe treatment; (c) an Eastern Cooperative Oncology Group performance status (ECOG PS) of 0 or 1; (d) classified as BCLC stage B beyond up‐to‐seven; (e) undergoing treatment with Atez/Bev or LEN; (f) a minimum 30‐d interval post‐TACE and before systemic therapy; (g) presence of at least two quantifiable lesions; and (h) absence of high‐risk factors for esophageal‐gastric variceal hemorrhage, as verified by endoscopy. Patients were excluded if they had received prior systemic therapy, were receiving Atez/Bev or LEN as secondary or tertiary treatment, or had other concurrent cancers.

### 2.3 Administration of systemic therapy

An interdisciplinary team determined the therapeutic regimens. The LEN dosage was tailored to the weight of each patient; a daily administration of 8 mg for patients weighing under 60 kg, whereas 12 mg for those weighing 60 kg or above. Atez/Bev treatment entailed a triweekly intravenous dose of 1200 mg atezolizumab and 15 mg/kg bevacizumab. Symptomatic management without dosage modification was advised for patients exhibiting mild‐to‐moderate adverse events (grades 1–2). For those presented with more severe toxicities (grades 3–4) in the LEN cohort, a reduced dosage or treatment hiatus was suggested based on the safety and tolerance status of the patient. The Atez/Bev dosage was kept constant wherever feasible, and treatment was interrupted only when the side effects became intolerable. The decision of the physician on whether to persist with the initial regimen or proceed to an alternative treatment line was based on disease progression, as ascertained during follow‐up.

### 2.4 Follow‐up protocol and evaluations

Initial HCC diagnosis relied on multiphasic contrast‐enhanced CT or MRI scans, in line with EASL guidelines. Such imaging was repeated 4–6 weeks after the initial treatment and then at 2–3 month intervals, along with concurrent laboratory tests, including the evaluation of alpha‐fetoprotein levels and complete blood counts, and the conduct of hepatic‐renal function tests, for assessing treatment response. Overall survival (OS) was defined as the duration from the first Atez/Bev or LEN administration to either death or the last checkup date (October 31, 2023). Progression‐free survival (PFS) was defined as the period spanning from treatment initiation to either tumor progression or mortality. The best overall response assessment, aligned with RECIST 1.1 and mRECIST criteria,^22^, ^23^ ranged from CR) to progressive disease (PD), as independently evaluated by two radiologists; any discrepancies were resolved by a third senior specialist. The duration of response (DOR) was calculated from the initial CR or PR to progression or death. Of note, PD classification encompassed (a) new intrahepatic growth or macrovascular encroachment, (b) novel extrahepatic metastasis, or (c) over a 20% increase in the cumulative minimal diameter of target lesions. Whereas, CR necessitated the disappearance of arterial phase enhancement across all target and nontarget lesions, nodes <10 mm, and normalized serum AFP levels. Adverse events were classified according to the Common Terminology Criteria for Adverse Events (CTCAE) version 5.0.

### 2.5 Statistical analysis

Data involving continuous variables are presented as either the mean ± standard deviation (SD) or the median ± interquartile range (IQR). Comparisons of these variables across the two treatment cohorts were performed using the Student's *t*‐test for parametric data and the Mann–Whitney *U*‐test for nonparametric data. Categorical variables are presented as percentages, with the differences between groups being assessed using the Pearson's *χ* ^2^‐test or Fisher's exact test as appropriate. The Wilcoxon paired signed‐rank test was used to evaluate changes in continuous variables from the initial measurement to subsequent follow‐ups. Survival outcomes, including OS, progression‐free survival (PFS), and DOR were estimated using the Kaplan–Meier method. The log‐rank test was used to determine the statistical significance of the differences in survival times. Subgroup analyses were conducted considering baseline demographic and clinical characteristics that influence survival. All statistical analyses were performed using GraphPad Prism (version 9.0; GraphPad Software, LLC, Boston, MA, USA). A *p* value below 0.05 indicated statistical significance.

## 3 RESULTS

**FIGURE 1:**
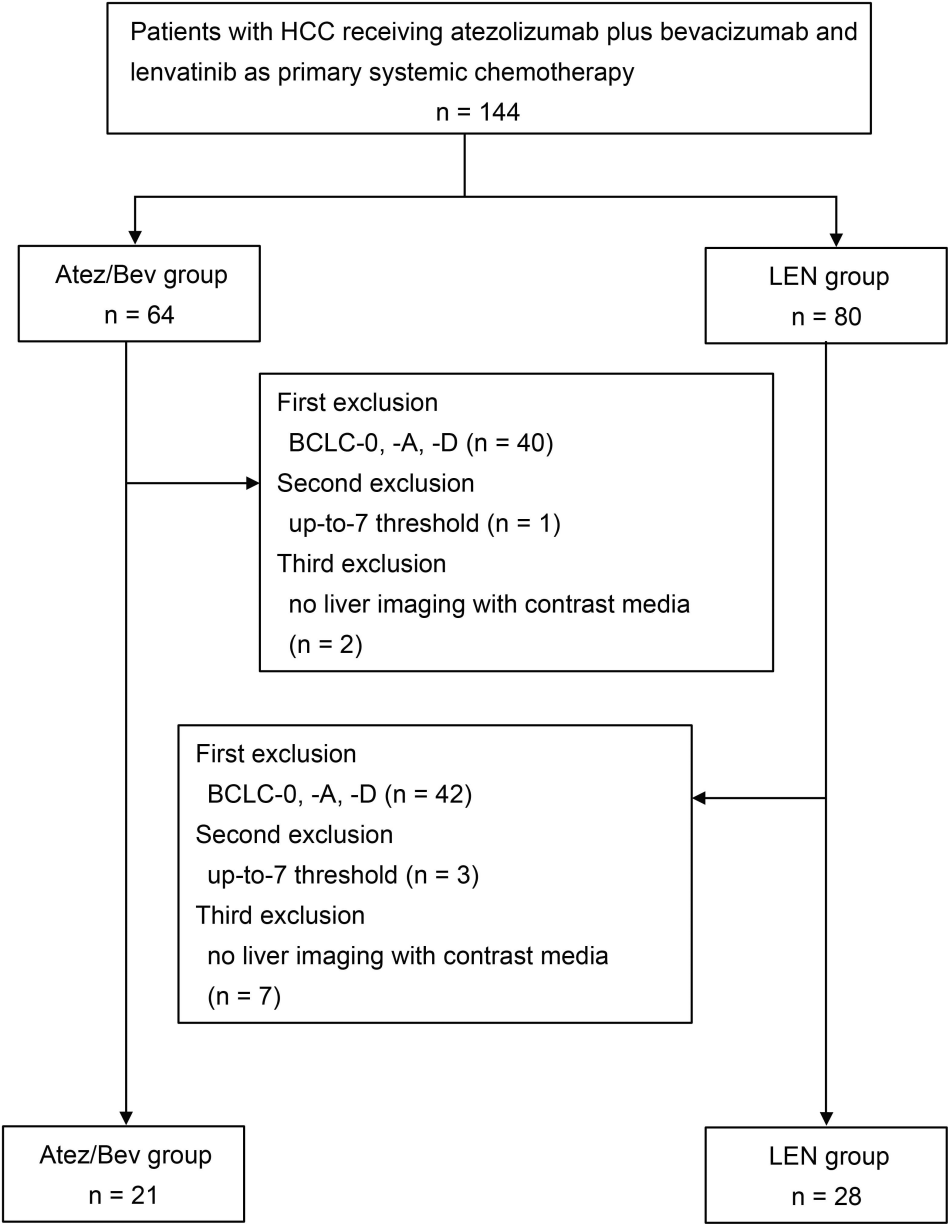
Patient Selection Flowchart. This figure illustrates the process of patient enrollment and selection for the study. A total of 144 patients with hepatocellular carcinoma (HCC) were initially considered for inclusion to receive either atezolizumab plus bevacizumab (Atez/Bev) or lenvatinib (LEN) as their primary systemic chemotherapy. After applying the inclusion criteria, 21 patients were allocated to the Atez/Bev group and 28 to the LEN group for the comparative analysis of treatment efficacy and safety.

**FIGURE 2:**
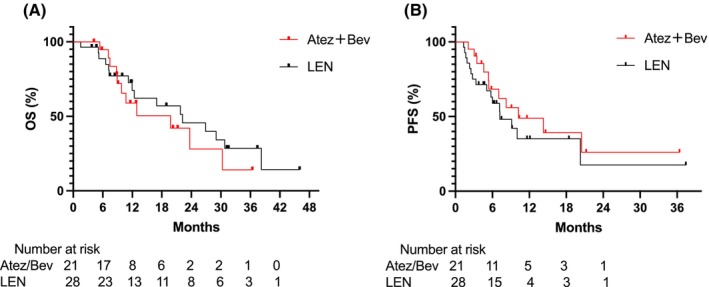
(A) Kaplan–Meier survival function for lenvatinib versus atezolizumab plus bevacizumab depicting overall survival (OS). (B) Kaplan–Meier survival function for lenvatinib versus atezolizumab plus bevacizumab depicting progression‐free survival (PFS).

A flowchart of the study is shown in Figure 1. Of the 49 consecutive patients included in the study, 21 were placed in the Atez/Bev group, whereas 28 were in the LEN group. The baseline patient characteristics are presented in Table 1. We did not observe any significant differences in baseline features between the two groups. At the start of chemotherapy, the Child‐Pugh grade in both groups was grade A, with 21 (42.9%) and 28 (57.1%) patients exhibiting Albumin‐Bilirubin (ALBI) grade 1 and grade 2, respectively. As of October 31, 2023, the median follow‐up time was 13.3 months (IQR 7.3–20.1) for the Atez/Bev group and 17.1 months (IQR 7.2–28.5) for the LEN group. Overall, we did not detect any significant difference in OS between the two groups, as shown in Figure 2A; the median OS rate was 19.80 months in the Atez/Bev group and 22.20 months in the LEN group (HR 1.31; 95% CI 0.59–2.91; *p* = 0.763).

**TABLE 1:** Patient background and clinical characteristics.

Baseline characteristics	Total (*n* = 49)	Atez/Bev (*n* = 21)	LEN (*n* = 28)	*p* Value
Age, years, median (IQR)	74.0 (70.0–77.0)	74.5 (69.5–79.0)	75.5 (70.0–81.0)	0.767
Gender, *n* (%)				
Men	36 (73.5)	14 (66.7)	22 (78.6)	0.515
Women	13 (26.5)	7 (33.3)	6 (21.4)	
ECOG performance status, *n* (%)				
0	48 (98.0)	21 (100)	27 (96.4)	1.000
1	1 (2.0)	0 (0)	1 (3.6)	
Etiology of HCC, *n* (%)				
Viral	20 (40.8)	5 (23.8)	15 (53.6)	0.045
Nonviral	29 (59.2)	16 (76.2)	13 (46.4)	
Child‐Pugh score, *n* (%)				
5	24 (49.0)	11 (52.4)	13 (46.4)	0.776
6	25 (51.0)	10 (47.6)	15 (53.6)	
ALBI grade				
1	21 (42.9)	11 (52.4)	10 (35.7)	0.263
2	28 (57.1)	10 (47.6)	18 (64.3)	
Prior treatment history, *n* (%)				
TACE	21 (42.9)	11 (52.4)	10 (35.7)	0.263
RFA	13 (26.5)	7 (33.3)	6 (21.4)	0.515
Surgery	5 (10.2)	1 (4.8)	4 (14.3)	0.376
History of Disease or Complication, *n* (%)				
Hypertension	37 (75.5)	11 (52.4)	16 (57.1)	0.779
Diabetes	20 (40.8)	8 (38.1)	12 (42.9)	0.777
Maximum tumor diameter, cm, median (range)	4.5 (1.8, 12.0)	4.2 (1.8, 12.0)	4.5 (2.1, 12.0)	0.486
Sum of diameters, cm, median (range)	11.2 (4.8, 37.9)	12.4 (4.8, 34.4)	11.1 (6.2, 37.9)	0.693
Number of tumors ≥6, *n* (%)	16 (32.7)	10 (47.6)	6 (21.4)	0.070
AFP, ng/mL, median, (range)	13.6 (1.9, 2066)	12.5 (2.6, 1009.1)	31.6 (1.9, 2066)	0.258
DCP, mAU/mL, median, (range)	2237 (10, 35,510)	2502 (17, 35,510)	2038 (10, 35,293)	0.799

As shown in Figure 2B, the median PFS was 10.23 months in the Atez/Bev group, whereas it was 7.20 months in the LEN group (HR 0.69; 95% CI 0.33–1.48; *p* = 0.343). As we did not observe any statistically significant difference in the OS or PFS between the two cohorts, we compared the best overall response between the two groups (Table 2). According to mRECIST standards, the objective response rate (ORR) and disease control rate (DCR) of the Atez/Bev group were 66.7% and 71.4%, whereas those of the LEN group were 46.4% and 75.0%, respectively (*p* values for ORR and DCR were *p* = 0.246 and *p* = 1.000, respectively).

**TABLE 2:** Summary of Best Overall Response.

Best Response	mRECIST		
	Atezo/Bev (*n* = 21)	LEN (*n* = 28)	*p* Value
Complete response (CR)	3 (14.3)	2 (7.1)	
Partial response (PR)	11 (52.3)	11 (39.3)	
Stable desease (SD)	1 (4.8)	8 (28.6)	
Progressive didease (PD)	4 (19.0)	6 (21.4)	
Evaluation Not possible (NE)	2 (9.5)	1 (3.6)	
ORR (CR + PR)	14 (66.7)	13 (46.4)	0.246
DCR (CR + PR + SD)	15 (71.4)	21 (75.0)	1.000

Abbreviations: Atez/Bev, atezolizumab plus bevacizumab; DCR, disease control rate; LEN, lenvatinib; ORR, objective response rate.

### 3.1 Adverse events

The common adverse events are summarized in Table 3. We observed that no treatment‐related deaths occurred in either group. We found that common adverse events of any grade in the Atez/Bev group mainly included elevated levels of aspartate aminotransferase (AST) (76.2% vs. 53.6%, *p* = 0.139) and alanine aminotransferase (ALT) (66.7% vs. 46.4%, *p* = 0.246), and hyperbilirubinemia (52.4% vs. 28.6%, *p* = 0.139) compared with those in the LEN group. Notably, the incidence of hypertension (38.1% vs. 28.6%, *p* = 0.547) and hand‐foot skin reactions (28.6% vs. 25.0%, *p* = 1.000) were not significantly different between the two groups. Due to severe adverse events, treatment was suspended in four patients (19.0%) in the Atez/Bev group, three of whom were due to malaise. In addition, we observed a 28.6% incidence of dose reduction or interruption in the LEN group.

**TABLE 3:** Safety Profiles and Adverse Events.

Adverse Event	Any Grade			Grades 3–4		
	Group, No. (%)			Group, No. (%)		
	Atez/Bev group (*n* = 21)	LEN group (*n* = 28)	*p* Value	Atez/Bev group (*n* = 21)	LEN group (*n* = 28)	*p* Value
Fever	4 (19.0)	2 (7.1)	0.381	0 (0)	0 (0)	1.000
Malaise	8 (38.1)	12 (42.9)	0.777	3 (14.3)	4 (14.3)	1.000
Anorexia	7 (25.0)	13 (46.4)	0.394	2 (9.5)	5 (17.9)	0.683
Diarrhea	4 (19.0)	8 (28.6)	0.517	1 (4.8)	3 (10.7)	0.625
Edema	6 (28.6)	10 (35.7)	0.760	2 (9.5)	3 (10.7)	1.000
Ascites	5 (23.8)	8 (28.6)	0.755	1 (4.8)	3 (10.7)	0.625
Elevated AST	16 (76.2)	15 (53.6)	0.139	2 (9.5)	1 (3.6)	0.569
Elevated ALT	14 (66.7)	13 (46.4)	0.246	2 (9.5)	1 (3.6)	0.569
Hyperbilirubinemia	11 (52.4)	8 (28.6)	0.139	2 (9.5)	1 (3.6)	0.569
Hypoalbuminemia	6 (28.6)	9 (32.1)	1.000	2 (9.5)	3 (10.7)	1.000
Proteinuria	6 (28.6)	11 (39.3)	0.549	1 (4.8)	1 (3.6)	1.000
Hypertension	8 (38.1)	8 (28.6)	0.547	5 (23.8)	3 (10.7)	0.263
Hand‐foot skin reaction	6 (28.6)	7 (25.0)	1.000	2 (9.5)	2 (7.1)	1.000
Gastrointestinal bleeding	2 (9.5)	2 (7.1)	1.000	0 (0)	0 (0)	1.000

Abbreviations: ALT, alanine aminotransferase; AST, aspartate aminotransferase; Atez/Bev, atezolizumab plus bevacizumab; LEN, lenvatinib.

## 4 DISCUSSION

For advanced HCC, Atez/Bev is recommended as the first choice of treatment, with LEN being suggested as an alternative option.^24^ However, the efficacy and side effects of Atez/Bev and LEN remain debatable, and only limited direct comparisons of these treatments are available. Notably, our study provided insights into the efficacy and safety of Atez/Bev versus LEN for patients specifically characterized as TACE‐refractory, that is, those with intermediate‐stage HCC and a tumor burden beyond the up‐to‐seven threshold who are unsuitable for TACE. We defined TACE‐refractory patients as those who were unlikely to benefit from TACE due to conditions that rapidly become refractory to TACE; those in whom TACE may deteriorate hepatic functional reserve to Child‐Pugh class B; or those with tumors resistant to TACE.^25^ In our study, the Atez/Bev and LEN groups showed comparable OS, PFS, and ORR, both demonstrating acceptable safety profiles. These results provide clinicians with important evidence for selecting either of these agents as the first‐line treatment for patients with intermediate‐stage HCC exceeding the up‐to‐seven threshold.

Recently, four retrospective real‐world studies investigated the efficacy and safety of Atez/Bev and LEN as first‐line treatments for advanced HCC.^26^, ^27^, ^28^, ^29^ In three of the four studies, similar OS was observed between the Atez/Bev and LEN groups.^26^, ^27^, ^29^ In the third study, Atez/Bev resulted in higher OS rates compared with those in the LEN group.^28^ In our study, the median OS rates were 19.80 months in the Atez/Bev group and 22.20 months in the LEN group, with no significant difference among them (HR 1.31; 95% CI 0.59–2.91; *p* = 0.763). Regarding PFS, our study also found no significant difference in the therapeutic effect between the LEN group (7.2 months) and Atez/Bev group (10.2 months) (HR 0.69; 95% CI 0.33–1.48; *p* = 0.343). Our findings were consistent with the findings of the South Korean and Taiwan studies, both of which reported no significant difference in median PFS, with the LEN group exhibiting 6.0 months and 5.9 months, respectively, and the Atez/Bev group showing 5.7 months and 5.3 months, respectively, suggesting comparable efficacy between the two treatments.^26^, ^29^ In contrast, the findings from two Japanese studies indicated that Atez/Bev administration resulted in a more favorable PFS (8.8 months) compared with that in the LEN group (5.2 months), further demonstrating higher PFS rates at half a year, 1 year, and one and a half years (56.6%, 31.6%, and not estimable, respectively) compared with those treated with LEN (48.6%, 20.4%, and 11.2%, respectively).^27^, ^28^ Regarding ORR, all five real‐world studies, including ours, showed no significant differences between the Atez/Bev and LEN groups. According to mRECIST standards, the ORR and DCR of the Atez/Bev group were 66.7% and 71.4%, whereas those of the LEN group were 46.4% and 75.0%, respectively (*p* values for ORR and DCR were *p* = 0.246 and *p* = 1.000, respectively).

Compared with the findings of the four other real‐world studies, this study showed more favorable OS and PFS outcomes, which may be partially attributed to the exclusive inclusion of patients with Child‐Pugh class A scores. However, it is important to note that similar proportions of Child‐Pugh class A patients have been commonly reported in real‐world studies. Therefore, the improved outcomes observed in our study are likely not explained by this factor alone. The particular advantage of our study was its focus on patients defined as TACE‐refractory, emphasizing the potential of early systemic therapy in a patient subgroup that may not typically benefit from TACE. This particular patient group is at a crucial juncture where early systemic chemotherapy can be highly beneficial, potentially preventing progression to stage C with poorer prognostic outcomes. Thus, our study underscored the importance of early intervention in a well‐defined patient population, suggesting that timely therapeutic strategies are essential for improving outcomes in HCC, beyond the effects of liver function classification alone.

Upon comparison of baseline characteristics, the Atez/Bev group mainly consisted of patients with nonviral liver diseases, whereas the LEN group had a higher proportion of patients with viral liver diseases.

Regarding the safety of treatment, LEN administration was associated with a similar incidence rate of treatment‐related adverse events (TRAEs) of any grade as Atez/Bev, with no significant differences in the occurrence of serious TRAEs being observed between the two regimens. Both regimens frequently resulted in increased levels of AST and ALT. However, these drug‐induced elevations rarely necessitated treatment discontinuation. Regarding serious TRAEs, anorexia and malaise were more common in the LEN group, whereas hypertension and malaise were more common in the Atez/Bev group. Overall, both LEN and Atez/Bev regimens were safe, with no unexpected adverse events.

Our study had several limitations. First, this was an observational study in which participants administered LEN and Atez/Bev were not simultaneously recruited. Specifically, patients treated with LEN were enrolled from May 2018 to October 2023, whereas those receiving Atez/Bev were recruited between October 2020 and October 2023. This difference in recruitment periods reflects the availability of these treatments in clinical practice, with Atez/Bev being approved after LEN. While we have accounted for several confounding factors in our analyses, we acknowledge that the nonsimultaneous recruitment could potentially introduce a selection bias. Second, the cohort size was limited, complicating additional stratified analyses.

In summary, the results of our study indicate that LEN offers similar efficacy and safety as Atez/Bev in the treatment of BCLC stage B HCC exceeding the up‐to‐seven benchmark as an initial therapeutic option. Although propensity score matching could have been used to reduce potential biases arising from this staggered recruitment, our sample size was not sufficiently large to employ this method effectively. Future studies with larger cohorts might overcome this limitation, potentially refining the comparative outcomes between the two treatment regimens.

## 5 CONCLUSIONS

Our study demonstrated that both Atez/Bev and LEN offer effective and safe first‐line systemic treatment options for patients with BCLC stage B HCC exceeding the up‐to‐seven threshold. These findings underscore the potential of both Atez/Bev and LEN as viable treatment alternatives for this particular patient subset. However, further validation of these results through extensive multicenter prospective studies is required.

## AUTHOR CONTRIBUTIONS

**Masamichi Kimura:** Conceptualization (lead); data curation (lead); formal analysis (lead); investigation (lead); methodology (lead); project administration (equal); resources (lead); software (lead); supervision (lead); validation (lead); visualization (lead); writing – original draft (lead); writing – review and editing (equal). **Koji Nishikawa:** Formal analysis (supporting); validation (supporting). **Jun Imamura:** Investigation (supporting); supervision (supporting); validation (supporting). **Kiminori Kimura:** Funding acquisition (lead); investigation (supporting); resources (supporting).

## FUNDING INFORMATION

No specific funding was received for this work.

## CONFLICT OF INTEREST STATEMENT

The authors declare that they have no conflict of interest.

## ETHICS STATEMENT

This study was approved by the ethics committee of the Tokyo Metropolitan Komagome Hospital (approval no. 2516) and adhered to the principles of the Declaration of Helsinki. The requirement for informed consent was waived owing to the retrospective nature of the study, which ensures no compromise to the welfare or rights of the patients.

## Data Availability

The datasets used and/or analyzed during the current study are available from the corresponding author on reasonable request.
